# Assessment of air quality in the Philadelphia, Pennsylvania subway

**DOI:** 10.1038/s41370-024-00711-9

**Published:** 2024-08-14

**Authors:** Anjum Shahina Karim, Maeve Malone, Alex Bruno, Aimee L. Eggler, Michael A. Posner, Kabindra M. Shakya

**Affiliations:** 1 Department of Geography and the Environment, Villanova University, Villanova, PA USA; 2 Department of Chemistry, Villanova University, Villanova, PA USA; 3 Department of Mathematics and Statistics, Villanova University, Villanova, PA USA

**Keywords:** Air Pollution, Exposure assessment, Indoor dust/house dust/dust, Inhalation Exposure, Particulate Matter

## Abstract

**Background:**

Subways are popular and efficient modes of transportation in cities. However, people are exposed to high levels of particulate matter (PM) in subways. Subway air quality in the United States has been investigated in a few cities, but data is lacking on simultaneous measurement of several pollutants, especially ultrafine particles (UFP) and black carbon (BC), in combination with different size fractions of PM.

**Objectives:**

The goals of this study are to assess air quality in a belowground subway and compare it with outdoor ambient levels, to examine temporal variability of PM in the subway, and to analyze the correlation between PM and BC.

**Methods:**

Particulate matter of varying sizes (PM_1_, PM_2.5_, PM_10_), UFP, and BC were measured using DustTrak, nanoparticle detector, and micro aethalometer, respectively. Measurements were made at the belowground subway platform and the aboveground street level at 15th Street subway station in Philadelphia during summer 2022.

**Results:**

Belowground mean PM_1_, PM_2.5_, and PM_10_ were 112.2 ± 61.3 µg/m^3^, 120 ± 65.5 µg/m^3^, and 182.1 ± 132 µg/m^3^, respectively, which were 5.4, 5.7, and 7.6 times higher than the respective aboveground street levels. The UFP lung deposited surface area (LDSA) (59.4 ± 36.2 µm^2^/cm^3^) and BC (9.5 ± 5.4 μg/m^3^) belowground were 1.7 times and 10.7 times higher than the aboveground. The pollutant concentration varied from day-to-day on both the locations. A higher positive correlation was found between the belowground BC and PM_2.5_ (*r* = 0.51, *p* < 0.05) compared to the aboveground (*r* = 0.16, *p* < 0.05).

**Impact:**

This study showed high levels of particulate matter exposure at a belowground subway station in Philadelphia. Particulate matter levels were about 5 to 8 times higher at belowground subway station than the corresponding aboveground street level. Higher levels were also observed for UFP lung deposited surface area (LDSA), while black carbon levels showed the highest concentration at the belowground level by a factor of ten compared to the aboveground level. The study shows the need for air quality management at belowground subways to reduce particulate matter exposure for the commuters.

## Introduction

Air pollutants have several adverse effects on human health, and particulate matter (PM) is one of the main concerns. Exposure to high PM levels can cause a wide range of cardiovascular effects including increased blood pressure, blood clotting disorders, cardiac rhythm alteration, coronary artery diseases, asthma, reduction in lung function and cardiovascular and cerebrovascular diseases [1–5]. Previous studies indicate that small particles, such as ultrafine particles (UFPs), can be more harmful to human health than large particles [6–10]. Given the smaller size of UFPs, the UFP number concentration along with LDSA (lung-deposited surface area) metrics are considered to be important determinants of toxicological impacts of the particles [11]. UFPs have a large reactive surface area and can penetrate deeper into the alveoli as well as the bloodstream [2, 12]. Higher exposure to UFP can increase allergic responses in human, airway inflammation, reduced expiratory flow, neurodegenerative diseases and even higher incidence of diabetes [13–16]. Black carbon is another component of PM, generated from combustion activities, and it also has adverse health effects upon inhalation, such as alteration in cardiovascular and respiratory functions [17, 18].

Subways are one of the best alternative modes of transportation in terms of both number of passengers carried per vehicle and low pollutant emission production [19]. However, the enclosed space of the belowground subway platform can expose people to higher concentrations of PM [20]. PM in the belowground subway platform can be generated from inside sources, such as the wear of the wheels, rails, electric cable abrasion, rail grinding, and ballast [21, 22]. In addition, belowground PM can be generated from outside sources such as fuel combustion, soil dust, resuspension of the particles from commuter movements, train piston effect, position of the tunnel ventilation shafts and mechanical fans [21, 23]. For UFP, the reported major sources in the belowground subway are from outdoor vehicle emissions and brake and tire dust, and indoor sources such as brake abrasion [13, 24, 25]. Black carbon (BC), which is one of the light-absorbing constituents of PM, can be generated from diesel-powered trains, used for the operation and maintenance of subway cars, usage of the graphite lubricant on the brake discs, and other materials inside the belowground platform, or from outside road traffic [26–28]. Due to the complex mixture of the PM in the belowground, and the reduced air mixing opportunity, the concentration of the pollutants in the belowground varies compared to the aboveground locations [29, 30].

Most studies on belowground subways focus on PM_2.5_ and PM_10_ concentrations [28, 31–34], and studies show the concentrations belowground are usually higher than the corresponding street levels in cities around the world. In Paris, France, the average daytime PM_2.5_ and PM_10_ concentrations in the underground railway station were approximately 5–30 times higher than Paris streets [35]. In London, United Kingdom, underground subway platforms had a much higher PM_2.5_ mass concentration (270–480 µg/m^3^) than the train cab concentrations (130–200 µg/m^3^) [36]. In Athens, Greece, PM_2.5_ and PM_10_ concentrations at an underground platform (88.1 and 320.8 µg/m^3^, respectively) were higher compared to both new (16.8 and 58.3 µg/m^3^, respectively) and old train cabins (47.5 and 238.8 µg/m^3^, respectively) [37]. In comparing PM_2.5_ concentrations among the underground subways of four cities of the northeastern United States (US), Luglio et al. [38] found the highest PM_2.5_ concentration in the underground subway environment of New York (547 µg/m^3^) followed by Washington D.C (341 µg/m^3^), Boston (327 µg/m^3^), and Philadelphia (112 µg/m^3^). In our previous study conducted at 12 underground subway stations in Philadelphia during the spring of 2018 and 2019, the PM_2.5_ concentration was found to be approximately 5.1 times and 2.6 times higher, respectively, than the corresponding aboveground street levels [34].

Southeastern Pennsylvania Transportation Authority (SEPTA) is the main transit provider for five counties in the Philadelphia region (Pennsylvania, US). An estimated 367,833 people used SEPTA subways for their daily commute in 2022 [39]. While studies have been published on the air quality of the subway environment in the US [26, 34, 38, 40], to our knowledge, no prior study has measured the levels of UFP and BC in combination with different size fractions of PM (PM_1_, PM_2.5_, PM_10_) at a US subway station. This study provides an assessment of air quality by measuring UFP and BC concentrations in combination with different size fractions of PM (PM_1_, PM_2.5_, PM_10_) at the busiest underground subway station in Philadelphia (15th Street), using 6 h collection windows. This study aims to investigate the exposure level of different size fractions of PM (PM_1_, PM_2.5_, PM_10_) in combination with UFP and BC by comparing both the aboveground and belowground locations of the busiest subway station of Philadelphia.

## Methods

### Sampling site

There are two main subway lines in the city of Philadelphia, Pennsylvania, United States: Market-Frankford line and Broad Street line [41]. In this study, the 15th Street subway station was selected, which is in the center of the city and intersects both the Market-Frankford line and Broad Street line. Our previous study found that the 15th Street station to have the highest pollutant (PM_2.5_, PM_10_, BC and CO_2_) concentration among the 12 underground subway stations in Philadelphia [34]. SEPTA’s Market-Frankford line as well as other subway-surface routes of trolley lines are linked to this underground station [41]. Additionally, 15th Street Station also serves the Broad Street subway line near City Hall with a belowground concord, which is further connected with Suburban Station for regional rails (Figs. S1 and S2). Because of its connection to several other subway lines, this station is considered the busiest subway station in the city of Philadelphia [41]. The sampling location for this study was located on the second floor belowground platform of 15th street station’s market Frankford line (which runs from east to west of the city) (39°57′10″N, 75°09′56″W) and had four adjacent tracks nearby. In the platform, there were 2 tunnels, separated by a wall in between, and each tunnel had two subway tracks.

Measurements from the corresponding aboveground street level (at the entrance of the same subway station) were also collected simultaneously (Fig. S2). As both sites are likely to be immediately influenced by local emission sources such as high traffic in the city, we also measured PM at one suburban location (on the roof of a four-story building at Villanova University) for the comparison. The distance from the 15th Street subway station to the suburban location is about 14 miles (23 kilometers) aerial distance (Fig. S1).

### Instrumentation

The DustTrak aerosol monitors (DRX model 8533, TSI Inc.) were used to measure different size fractions of PM (PM_1_, PM_2.5_, PM_10_), and nanoparticle detectors (Nanos Partector 2 model, Naneos) were used for measuring UFP particle number and lung deposited surface area (LDSA). The instruments were newly purchased and had been calibrated by the manufacturer. According to the manufacturer, the DustTrak monitors were calibrated to A1 Test Dust (size distribution between 0.1 to 10 µm with particle density of 2.65 g/cc). The zeroing of DustTrak was done prior to measurement following the instrument’s manual. Micro aethalometers (microAeth AE51 model, Aethlabs Inc.) were used to measure BC. Some studies mentioned referring BC as equivalent BC (eBC) [42], however, in this study, we decided to refer to the light absorbing properties of the particles as BC. A new filter strip was changed prior to each measurement. The raw data from the micro aethalometer (BC raw data) was further processed using Optimized Noise Reduction Averaging (ONA) method [43]. Data for pollutant concentrations were logged at 10 s intervals except for UFP (1 s interval). Since the UFP interval was different from other pollutant logging, the data from UFP was merged with PM and BC using R software. Temperature and humidity were measured using the HOBO MX (MX1101) with fifteen-minute intervals. Since two sets of each instrument were used for concurrent measurements from the locations, prior to the sampling, a test run for all the instruments was made ensuring there was no discrepancy between the results from each set of instruments. Air monitoring instruments were placed facing the platform and the street respectively on both belowground and aboveground locations. In both the belowground and aboveground locations, the inlets of the instruments were placed at a height at about 45 inches (114 cm) above ground level.

Although the DustTrak instrument was recently calibrated, the measurement can be subject to bias based on the type of particles. To evaluate this bias, we collected PM_2.5_ particles on 47 mm PTFE filters (3 filters from each location for 5 days) with a SKC PM_2.5_ IMPACT sampler connected to a Leland Legacy Pump at a flow rate of 10 L/min. The IMPACT sampler contains a removable filter cassette and disposable pre-oiled impaction discs that were changed for each of the sampling period. Particles were collected for 6 h each from both locations. Prior to particle sampling, the filters were preconditioned in a chamber (temperature: ~23 °C and relative humidity: 30–50%) for 24 h and then were pre-weighed using a microbalance (XP2U model, Mettler Toledo). Details on comparison against gravimetric method is included in a supplementary section. Three replicate weight measurements were taken with a difference not greater than 1 µg between the three measurements. The difference of the relative humidity of the environment between pre and post weighing period is likely to be <10%. To prevent the electrostatic charges from the filters, the filters were passed through an antistatic stabilizer (Mettler Toledo U-electrode) before each filter weighing. After the particle collection, filters were placed again in the conditioning chamber for 24 h prior to post-weighing. We present the evaluation of DustTrak measurements in the supplementary section. Although bias was found by DustTrak measurements against the gravimetric method, there was also variation within gravimetric method, and variability of bias. Therefore, we presented uncorrected PM measurements from gravimetric method, and report bias from DustTrak measurements against gravimetric method.

### Sampling schedule

Sampling was conducted from the 15th Street subway station continuously for 6 h from about 9 a.m. to 3 p.m. on 5 different days between Monday and Friday during July 13, 15, 18, 20 and 22, 2022. The sampling was conducted during the same month with small range of temperature and humidity conditions. The reason for choosing 9 a.m. to 3 p.m. for sampling was due to getting a long duration of sampling to collect the particles in the filters for performing cellular assay which is one of the main objectives of this project, and results from which will be presented elsewhere. Measurements from the suburban location were conducted at the roof of a four-story building on the Villanova University from June 22–24, 2022.

### Data analysis

PM concentrations that showed extremely high sudden increases were considered as instrumental errors and five data points were removed from all summary statistics, analyses, and graphical representations. Five data points constituted less than 0.06% of the total data.

All numerical measurements are reported as mean±one standard deviation (Table S1). The overall mean±one standard deviation was measured by combining the five days of data together. The ratio of pollutants belowground to aboveground was computed by using the mean concentration from each day. Welch’s two sample *t*-test was conducted to check for significant differences of each pollutant concentration between the two locations (belowground vs aboveground and aboveground vs suburban). All tests used a two-sided alternative and a significance level of 0.05 to determine statistical significance using R software.

## Results and discussion

### Pollutant concentrations at the belowground subway station

**Fig. 1:**
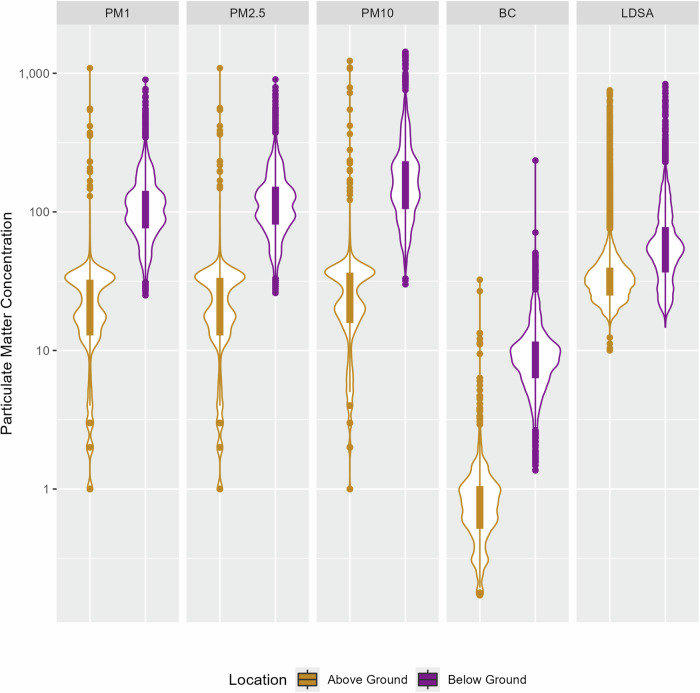
PM, BC, and UFP LDSA concentrations at aboveground (AG), belowground (BG). The box shows the interquartile range with median value for each location, whereas the violin pattern outside shows the entire pollutant distribution. The y-axis is presented in a log scale with units µg/m^3^ for PM_1_, PM_2.5_, PM_10_ and BC, µm^2^/cm^3^ for UFP LDSA. The dots show the outliers near both above and below the whiskers.

The belowground location had the highest PM concentrations compared to both the aboveground and the suburban location (Fig. 1 and S4). Mean belowground concentrations of PM_2.5_ and PM_10_ at 15th Street subway station were 120 ± 65.5 and 182.1 ± 132 µg/m^3^, respectively, and aboveground concentrations from the same street location were 22.2 ± 13.9 and 26.0 ± 15.7 µg/m^3^, respectively (Fig. 1, Table S1). At the suburban location, the PM_2.5_ and PM_10_ concentrations were much lower, 11.0 ± 5.6 µg/m^3^ and 11.7 ± 6 µg/m^3^, respectively, measured during the previous month (Fig. S4). The mean concentrations of UFP LDSA and BC were also higher belowground (59.4 ± 36.2 µm^2^/cm^3^ and 9.5 ± 5.4 µg/m^3^) respectively, compared to the respective aboveground 35.8 ± 21.7 µm^2^/cm^3^ and 0.9 ± 0.8 µg/m^3^ (Table S1).

Belowground at the Philadelphia 15th Street station, the PM_2.5_ mean concentration (120 ± 65.5 µg/m^3^) was higher than other cities’ subway stations such as Singapore (24.1 µg/m^3^) [44], Munich, Germany (27 to 80 µg/m^3^) [45], Athens, Greece (88.1 µg/m^3^) [37], and Stockholm, Sweden (76 µg/m^3^) [46]. While the mean PM_10_ concentration (182.1 ± 132 µg/m^3^) in Philadelphia was higher than a few cities, such as, Singapore (26.9 µg/m^3^) [44], some cities have higher belowground mean PM_10_ concentrations than Philadelphia, such as Tianjin, China (162.82 ± 25.6 µg/m^3^) [47], Athens, Greece (320.8 µg/m^3^) [37], Seoul, South Korea (359 µg/m^3^) [48], and Stockholm, Sweden (237 µg/m^3^) [46]. The BC concentration found belowground (9.5 ± 5.4 μg/m^3^) was also higher than some of the other subways reported previously, such as, Hongkong (3.6 ± 2.1 μg/m^3^) [49], Singapore (2.4 ± 1.3 μg/m^3^) [50], and Toronto (7.8 ± 7.2 μg/m^3^) [51], while lower than a few stations such as, New York (22.4 ± 7.5 μg/m^3^) [38]. Since limited studies has been done on UFP (LDSA) in the subway microenvironment, the number concentration of UFP, in few cities were also found to contain higher concentrations such as Beijing, China (13,245 ± 2936 particles/cm^3^) [10], than this study in this Philadelphia subway (17505 ± 8685 particles/cm^3^).

Based on data collected in February and August of 2015, Luglio et al. [38] found an overall mean concentration of PM_2.5_ in Philadelphia of 112 ± 46.7 µg/m^3^, which is similar to our present findings. In our previous study, [34] we found similar values at 15th Street station, with mean PM_2.5_ and PM_10_ concentrations of 110 ± 33 and 127 ± 33 µg/m^3^, respectively, in spring 2018, and 88 ± 44 and 109 ± 57 µg/m^3^, respectively, in spring 2019. Higher PM concentration at the 15th Street Philadelphia subway station than other subway systems may be because of inadequate ventilation and cleaning, and old age. High PM_1_ concentration also indicates an increased concern for adverse health consequences.

**Fig. 2:**
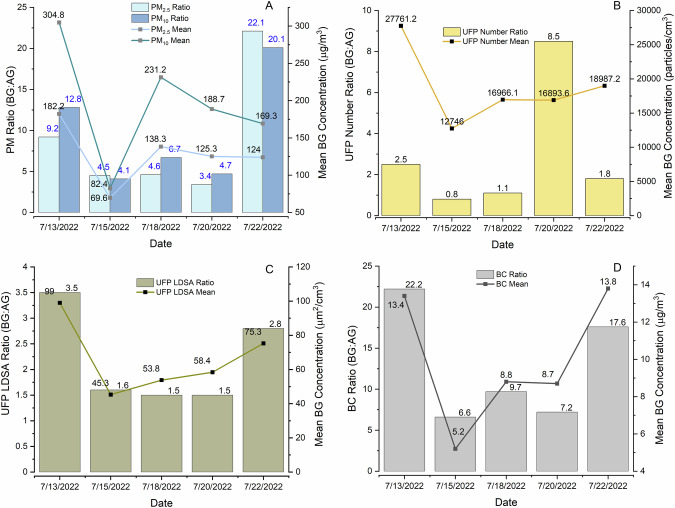
Belowground to aboveground ratio of PM_2.5_, PM_10_, UFP (number concentration and LDSA), and BC for all sampling days in bar graph. In the secondary y axis, respective mean pollutant concentration at belowground (BG) is shown with the point-line. Ratio (bar graphs) and mean (line graphs) of PM_2.5_ and PM_10_ are shown in (**A**). Similarly, the ratio and mean of UFP number concentration, LDSA and BC are shown in (**B**), (**C**), and (**D**), respectively.

Compared to the aboveground, the total mean concentrations for PM_2.5_ and PM_10_ in the belowground were 5.7 and 7.6 times higher, respectively (Fig. 2A). For three out of five days of measurement (Fig. 2A), the belowground to aboveground ratio for PM_2.5_ was smaller compared to that for PM_10_. This indicates that additional PM sources such as resuspension, brake dust besides combustion source at belowground. For UFP, both the LDSA and number concentration belowground were nearly 1.7 and 1.2 times higher, respectively than the aboveground (Fig. 2B, C). UFP (LDSA and number concentration) shows the smallest difference between aboveground and belowground compared to PM and BC. This indicates the minor UFP sources at belowground compared to that for PM and BC. BC concentration belowground showed the highest difference with 10.7 times higher than aboveground (Fig. 2D). In the absence of major combustion sources belowground, it is unlikely that all BC particles were from incomplete combustion sources. It is likely that some iron particles were measured as black carbon. Though there was a variability between the belowground to aboveground ratios of all the pollutants, the pattern for the variability for all the pollutants was similar in terms of the days (Fig. 2). On July 13th, all the means and ratios were higher than on all other days (except for the PM), whereas for July 15th, means and ratios were the lowest (Fig. 2). It is noteworthy to mention that for all the pollutants, when compared to all the other days, the belowground mean pollutant concentration was significantly lower on July 15th, 2022 (Table S1), whereas there was not very significant change in the mean in the aboveground concentration. One possible explanation for this lower concentration in the belowground on July 15th can be some cleaning activity that might have taken place in belowground (such as cleaning of the belowground floors or train tracks) between July 13th to 15th, 2022.

Both the UFP and BC are products from incomplete combustion and other combustion activities [49, 52–54], however, other natural and anthropogenic sources such as heating from the building or the power supply can be a potential source for UFP [13, 55]. Due to the diverse sources, the chemical composition of UFP can be from elemental and organic carbon, or inorganic components such as metals [13, 55]. Higher air dispersion can result in higher UFP concentration in the belowground from outdoor sources. However, the enclosed belowground space with higher UFP concentration indicates other indoor sources such as train frequency, friction of the metal wears, and wheels, electric power supply, crustal particles or even the efficiency of the mechanical ventilation system [13, 53]. A higher belowground BC concentration than the aboveground also indicates less opportunity for dispersion at belowground. There could also be BC originating from equipment for maintenance and cleaning in the subways. Additionally, the aethalometer measures BC based on the light absorption of particles. Therefore, the belowground, aethalometer may detect both soot from the combustion process and also responding to the light absorbing particles such as iron (Fe) particles [56, 57] generated from the wear of the wheels and tracks from the subway cars. Lyu & Olofsson [27] found that the use of graphite lubricant on the brake discs can generate BC. A study conducted in New York City found the BC concentration to be higher (5–23 μg/m^3^) in the belowground subway than at the street level (3 μg/m^3^) [26]. In another study of subways in four Northeastern cities (Boston, New York, Philadelphia and Washington D.C), BC in the belowground subway ranged from 5–12 μg/m^3^, and the highest level of 22.4 ± 7.5 μg/m^3^ was found in New York City [38]. The authors attributed high BC concentration to the diesel operated cars used in the underground stations at night [38]. Vilcassim et al. [26] found that depending on the station depth and line, BC concentration indicated high spatial variation. Since belowground sampling location was two floors below the street level (Fig. S2), the distant outdoor platform may have limited the air dispersion in the belowground. Thus, there was likely little circulation and mixing of air with outdoor air, suggesting perhaps the primary source of the BC was graphite lubricant on disc brakes and the diesel operated cars used for the maintenance of the subways. However, due to limited available information on the belowground maintenance protocol, mechanical ventilation system of the station, and possible overestimation of measurement artifacts, the sources of the belowground UFP and BC cannot be attributed confidently.

Regarding the overall high concentrations of PM than aboveground, the outdoor polluted air after entering the belowground subways is less likely to be removed by natural ventilation alone. Therefore, removal of belowground pollutants relies more on the mechanical ventilation system. High concentrations of PM in the belowground result from the abrasion of the wheels with the rail tracks and wires, train piston effects, and/or the resuspension of PM from the movement of both the commuters and the trains [34, 58, 59]. The station design, ventilation system, and the age of the infrastructure also play important roles in controlling the belowground air quality. Since this station was built in 1940 [41], the old infrastructure could be another contributing factor for the high level of belowground PM concentration. Hwang et al. [60] found the subway stations built before the 1970s in Seoul, South Korea had higher PM concentration than the stations that were built after the 1970s.

### Temporal variability between aboveground and belowground locations

**Fig. 3:**
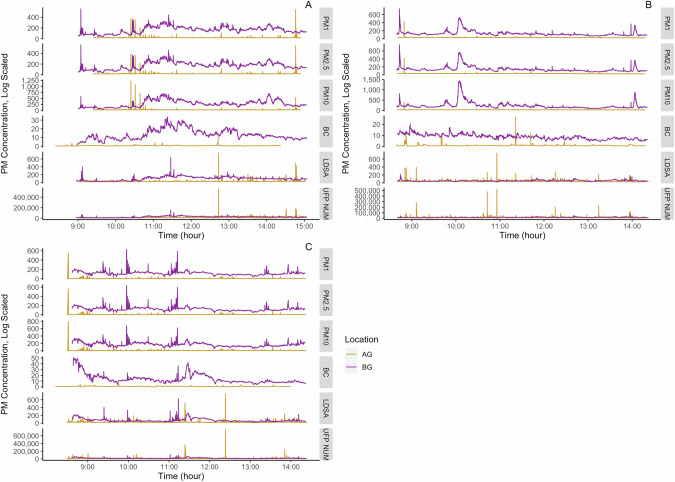
Patterns of PM concentrations for aboveground (AG) and belowground (BG) locations. **A** July 13, **B** July 18, and **C** July 22, 2022. The figure is presented in a log scale with units µg/m^3^ for PM_1_, PM_2.5_, PM_10_ and BC and for µm^2^/cm^3^ for UFP LDSA and particle/cm^3^ for number concentration.

The pollutant concentrations varied from day to day at belowground and aboveground location (Fig. 3). There was a greater variation of PM (PM_1_, PM_2.5_, PM_10_) and BC at the belowground than the aboveground location (Fig. 3).

**Fig. 4:**
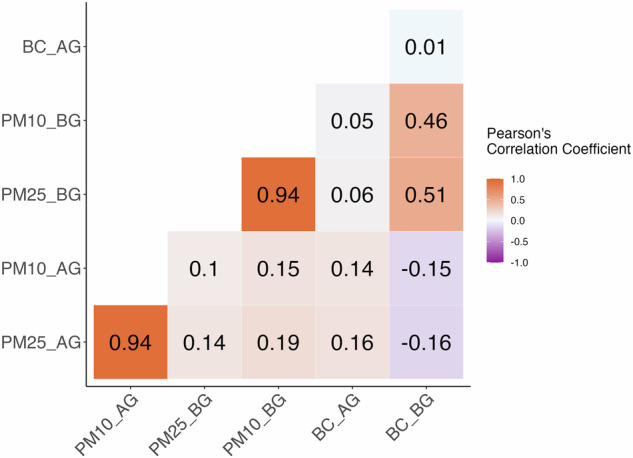
Correlation between aboveground (AG) and belowground (BG) black carbon (BC) and particulate matter. PM25 and PM10 in the figure represent PM_2.5_ and PM_10_, respectively.

Since the belowground location had two tunnels, with each tunnel having 2 subway tracks, the train frequency at this station was about every two to six minutes, causing spikes in the pollutant concentrations with arrivals and departures of trains. For all five days of measurements, BC and UFP concentrations in the belowground showed parallel rise with the aboveground concentration (Figs. 3 and 4) and a possible reason could be polluted air was also being carried out to the tunnel from the aboveground traffic activity [61]. As the primary source of BC and UFP is incomplete combustion activities, it is likely these particles entered the belowground location from aboveground traffic. Yang et al. [49] reported that the UFP number concentration near the busy city roads of Hong Kong was >100,000 particles cm^−3^, but there was less UFP number concentration in the platform (0.9 ± 0.5 × 10^4^ particles cm^−3^). In Rome, Italy, the subway systems with shallow underground stations with respect to road surface had greater UFP particles than the subway systems with deeper underground stations [6].

However, for PM, the belowground concentration fluctuations did not always correspond with the aboveground, meaning the belowground PM concentration can be generated from both the outdoor and indoor sources. Bendl et al. [45] found the frequency of trains to the platform had a significant effect on the belowground PM concentration. Additionally, Bendl et al. [45] found that after departure of two trains in opposite directions on adjacent tracks simultaneously, there were noticeable spikes on the PM concentrations for a short time. Given the complex structure of the station with adjacent two tunnels, with four tracks in opposite directions, the station experiences highly variable timings, dependent on train destinations. This variability leads to frequent trains entering and leaving the belowground location, potentially causing sudden spikes in pollutant concentrations.

### Correlation of PM and BC

Correlations tests were conducted between PM and BC to assess the combustion related PM. Correlation coefficients of BC and PM (PM_2.5_, PM_10_) were higher at the belowground than at the aboveground location (Fig. 4). This is different than what we expected considering the high combustion related sources such as traffic activity at aboveground busy street. This may further indicate that aethalometer is responding to iron-related particles and PM generated at belowground is related to iron particles generated from the train tracks. At the belowground, the correlation of BC with PM_2.5_ and PM_10_ was moderate (Fig. 4). Correlation of BC was slightly higher with PM_2.5_ (*r* = 0.51, *p* < 0.05) compared to PM_10_ (*r* = 0.46, *p* < 0.05) (Fig. 4). Whereas at the aboveground, the correlation of BC with PM_2.5_ (*r* = 0.16, *p* < 0.05) and PM_10_ (*r* = 0.14, *p* < 0.05) was much lower compared to the belowground (Fig. 4). Furthermore, the correlations between belowground PM_2.5_ and PM_10_ with the respective aboveground PM_2.5_ and PM_10_ show positive but weak correlations (*r* = 0.14, *p* < 0.05 and *r* = 0.15, *p* < 0.05) (Fig. 4). Besides outside traffic sources, factors such as frequency of train, commuter movements, and the particles generated from other sources (e.g. silicon from crustal materials [38], iron from frictions between wheels, rails, collections shoes and third rail [38], ambient soil particles from the tunnels [62]) can influence particle concentration at belowground.

In a study on the exposure to PM_2.5_ and BC among different subway workers in South Korea, Choi et al. [63] found a strong correlation between PM_2.5_ and BC (*r* = 0.72) for subway train drivers in terms of job exposure, followed by the maintenance engineers (*r* = 0.61) and subway station managers (*r* = 0.4). In explanation of their findings, Choi et al. [63] mentioned, for the maintenance engineers and station managers, the varying type of the tasks and worksites can be significant factors for the overall exposure to PM_2.5_ and BC. The maintenance engineers are exposed to the highest concentration of PM_2.5_ and BC while working on the repair and maintenance of the belowground tunnels at night after their completion of other day-to-day activities depending on the needs. However, the train drivers are constantly exposed to the PM_2.5_ and BC while operating the subway trains for a longer period.

In comparing different subway stations, Luglio et al. [38] also found a strong correlation between belowground BC and PM_2.5_ (*r* = 0.6). In this study, we found that the correlation between PM_2.5_ and BC belowground was similar to the previous studies (*r* = 0.51, *p* < 0.05). This high correlation of BC and PM belowground suggests a similar source of PM such as combustion and iron-related particles. When a train enters the station, it may bring a draft of outside air along with these pollutants, but, unlike the outside environment, there is less opportunity for dispersion of polluted air belowground. While comparing different subway lines in New York City, Vilcassim et al. [26] found that the stations with a shorter distance between the belowground and aboveground had lower BC concentration than the stations with longer distance. Li et al. [64] found BC was higher at the platform than in the train cabs, and upon the train arrival, BC increased which showed the source of BC was from the piston effect of the train. The piston effect is a phenomenon generated by a train moving in a confined tunnel with a high-speed which creates air pressure in front of the train and decreases the air pressure behind the train. In short, the piston effect of the subway can bring pollutants to the belowground generated from outside sources. Vilcassim et al. [26] mentioned that the BC found in the belowground are not necessarily freshly emitted and can be re-entered by rapidly passing trains. Due to the structure of the sampling location, the higher concentration of BC belowground also relates to the passage of trains bringing particles emitted from combustion sources aboveground.

### Limitations

Due to logistical limitations for conducting a long day of sampling, the sampling window excluded the extreme rush hours. One of the objectives of this study was to assess the cellular assays on particles collected, and it requires longer duration of sampling. The results from cellular assays will be presented on a separate paper. This data also represents only the conditions during weekdays. Data from weekends would further establish the trends of the pollutant concentration for the locations. Due to the variability in both the gravimetric and real-time measurements as well as the variability on day-to-day measurements, we did not use the correction of PM from dusttrak based on gravimetric measurement.

### Recommendations

Exposure of PM (PM_1_, PM_2.5_, PM_10_), UFP, and BC belowground were higher than the aboveground location and the suburban location. High PM concentrations (>100 µg/m^3^) may pose health risks to the travelers and workers at belowground subway stations. Therefore, control strategies are recommended to target reducing PM levels. Because of the confined structure in the belowground station, the trapped air has reduced opportunity to mix with clean and outside air. Improved and frequent mechanical ventilation can enhance such mixing. Implementing regular cleaning schedules for subway platforms can remove dust, litter, and debris from the tunnel walls as well as the belowground [28, 65] thus reducing the resuspension of these particles in air. Most importantly, frequent air circulation from the ventilation system along with regular cleaning of tracks and walls of the tunnels can significantly improve the air quality by reducing the resuspension of particles [58]. Enforcement of strict smoking bans and reducing passenger idling time within subway stations and platforms can also reduce the pollutant concentration in the underground space. In addition, installation of platform screen doors and HEPA filters in the ventilation can reduce pollutant exposure to the commuters and the workers. Martins et al. [58] reported in their study, installation of platform screen doors and separation of the rail track from the platform of a single tunnel station can reduce the belowground pollutant concentration by around 50% compared to the conventional systems (without platform screen doors). Since subways provide important services by reducing vehicle emissions and carrying a large number of passengers at a time, regular air quality monitoring of the underground stations can also help to check state of the indoor air quality, assess the factors/sources affecting air quality, and measure the effectiveness of future control strategies on improving air quality at underground subways.

## Conclusion

This study offers data on various size fractions of PM in combination of UFP and BC concentrations from the busiest 15th Street subway station of Philadelphia. The high ridership and the connections to other subway lines and trolley routes makes the station suitable for portraying the overall commuter exposure to these air pollutants. Since our sampling campaign offered concurrent measurements from above and below the station as well as from a suburban location, the pollutants measured offered comparable air quality data from a range of microenvironments. Our dataset showed the air pollutant (PM_1_, PM_2.5_, PM_10_, UFP LDSA, BC) concentrations belowground were several times (5.4, 5.7, 7.6, 1.7 and 10.7 times, respectively) higher than the adjacent aboveground location, which indicates that the belowground air quality can depend on not only the outside traffic, but also several factors including station design, age and depth of the station, abrasion of the train wheels and tracks, commuter density, resuspension of the particles, access to open air and overall ventilation process. Another finding suggested that although there is no major black carbon source inside the belowground subway station, the highly elevated BC concentration indicated reduced opportunity of air dispersion, as well as possible generation of iron-related particles from frictional activities. High correlation of BC with PM_2.5_ (*r* = 0.51, *p* < 0.05) and BC with PM_10_ (*r* = 0.46, *p* < 0.05) belowground indicates similar sources such as outside road traffic, graphite lubricant usage on the brake pads, a possible usage of diesel operated cars which are used for the maintenance of the subway cars, and iron-related particles. Since many commuters take the subway for their everyday commute and the workers in belowground subway stations are exposed to the pollutants for even a longer period, regular monitoring and maintenance are needed to improve the overall indoor air quality belowground. Installation of platform screen doors, improvements in the ventilation system, and increased frequency in cleaning the tunnel walls and floors can reduce the exposure to these harmful pollutants as well as minimize the health risk of both the workers and commuters.

## Supplementary information

Supplementary Information

## Data Availability

Data is available upon request.
