# Supplementary material for: Assessment of air quality in the Philadelphia, Pennsylvania subway
Source: J Expo Sci Environ Epidemiol. 2024 Aug 14;35(2):196–204. doi: 10.1038/s41370-024-00711-9 (PMC12009735; doi:10.1038/s41370-024-00711-9)
Supplement: Supplementary file 1 — Supplementary Information [file 41370_2024_711_MOESM1_ESM.docx]

**Supplementary Information**

**Assessment of air quality in the Philadelphia, Pennsylvania subway**

Anjum Shahina Karim^1^, Maeve Malone^1^, Alex Bruno^1^, Aimee L. Eggler^2^, Michael A. Posner^3^, Kabindra M. Shakya^1*^

# **^1^**Department of Geography and the Environment, Villanova University

# **^2^**Department of Chemistry, Villanova University

# **^3^**Department of Mathematics and Statistics, Villanova University

*Corresponding author

Kabindra M. Shakya

# Department of Geography and the Environment, Villanova University

800 E Lancaster Avenue

Villanova, PA 19085, USA

Phone: 610-519-3590

Email: [kabindra.shakya@villanova.edu](mailto:kabindra.shakya@villanova.edu)

The supplementary section contains information related to methods and results. The following information is included.

**List of Figures**

Figure S1. Sampling locations in the black box (map is modified from the SEPTA’s station map)

Figure S2. Sampling locations (aboveground and belowground)

Figure S3. Correlation of PM_2.5_ measurement between gravimetric and DustTrak aerosol monitor for (A) aboveground (r= 0.58) and (B) belowground (r= 0.83). Circles represent the mean concentration. For the DustTrak aerosol monitor, the error bars represent 1 standard deviation of six hours of measurement period. For the gravimetric method, the error bars represent 1 standard deviation of three replicate filter measurements. Three separate filters were measured concurrently at the same location. The dotted line represents the line of equality whereas the trendline is represented by the solid line.

Figure S4. PM, BC, and UFP concentrations at aboveground (AG), belowground (BG), and suburban location (ROOF). The box shows the interquartile range with median value for each location, whereas the violin pattern outside shows the entire pollutant distribution. The figure is presented in a log scale with units µg/m^3^ for PM_1_, PM_2.5_, PM_10_ and BC, µm^2^/cm^3^ for UFP LDSA, and particle/cm^3^ for UFP number concentration (UFP#). The dots show the outliers near both above and below the whiskers.

**List of Tables**

Table S1. Pollutant concentration at belowground (BG) and aboveground (AG) locations for both separate sampling days and all sampling days together during July 2022

Table S2. Real-time and gravimetric mean for PM_2.5_ with temperature and humidity for five different sampling days for aboveground and belowground

# Supplementary Method section

# Sampling Location


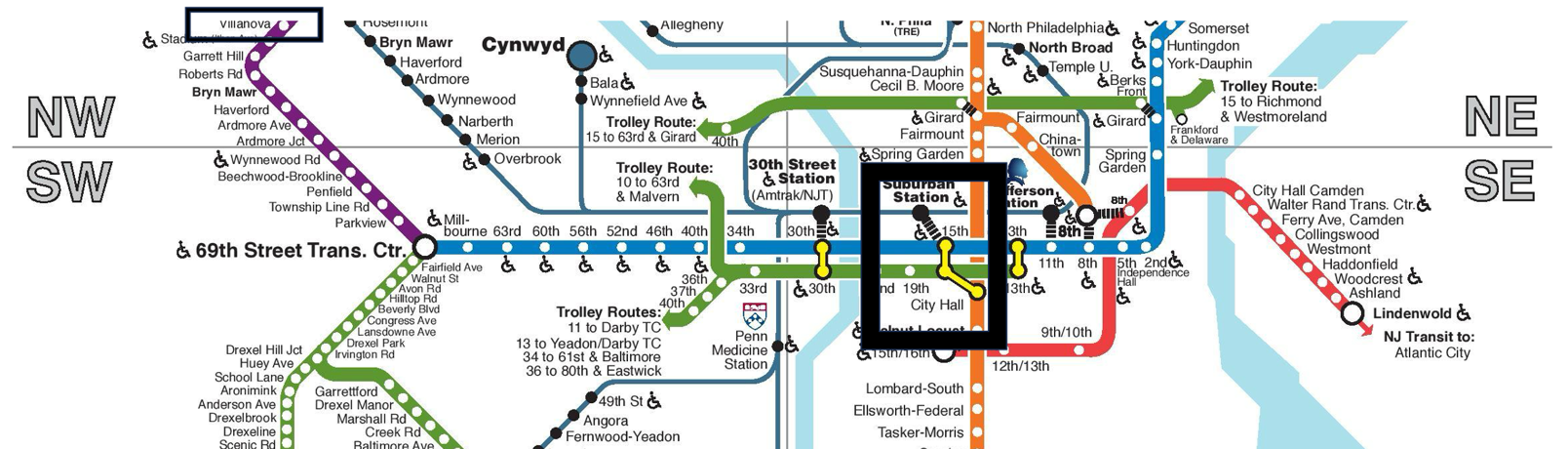


Figure S1. Sampling locations in the black box (map is modified from the SEPTA’s station map)


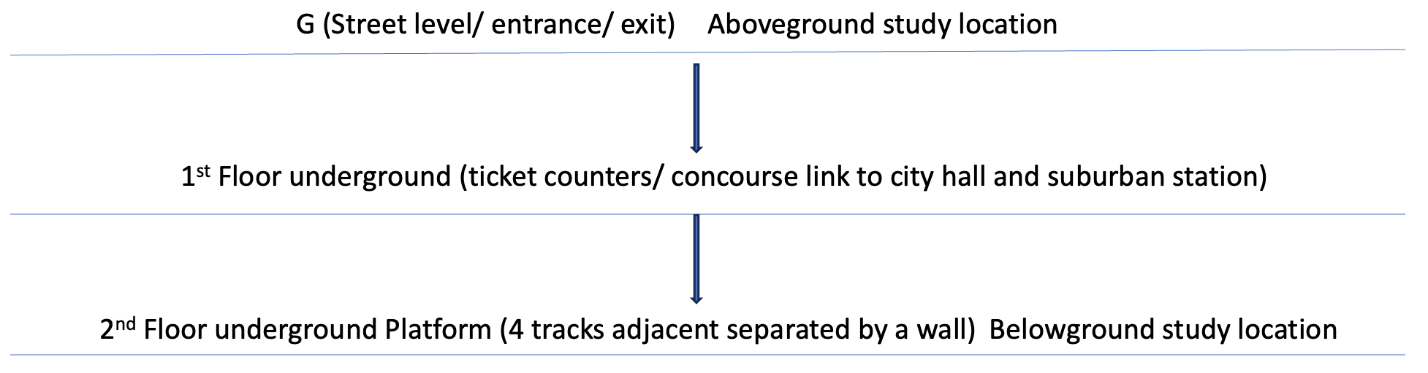


Figure S2. Sampling locations (aboveground and belowground)


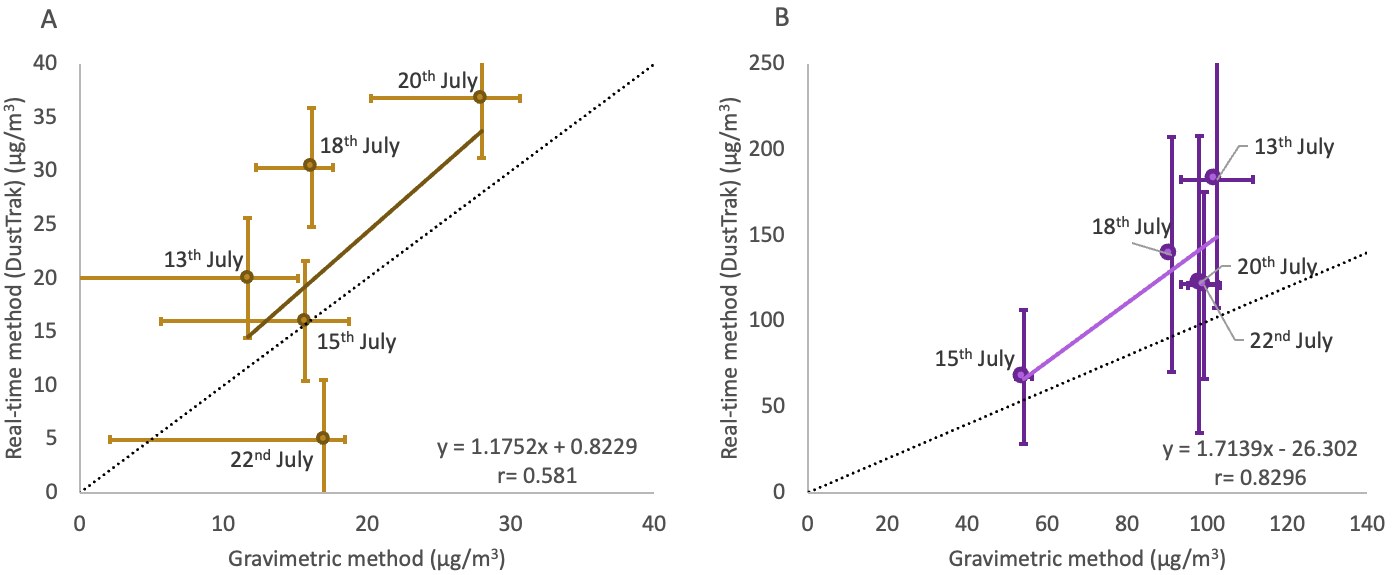


Figure S3. Correlation of PM_2.5_ measurement between gravimetric and DustTrak aerosol monitor for (A) aboveground (r= 0.58) and (B) belowground (r= 0.83). Circles represent the mean concentration. For the DustTrak aerosol monitor, the error bars represent 1 standard deviation of six hours of measurement period. For the gravimetric method, the error bars represent 1 standard deviation of three replicate filter measurements. Three separate filters were measured concurrently at the same location. The dotted line represents the line of equality whereas the trendline is represented by the solid line.


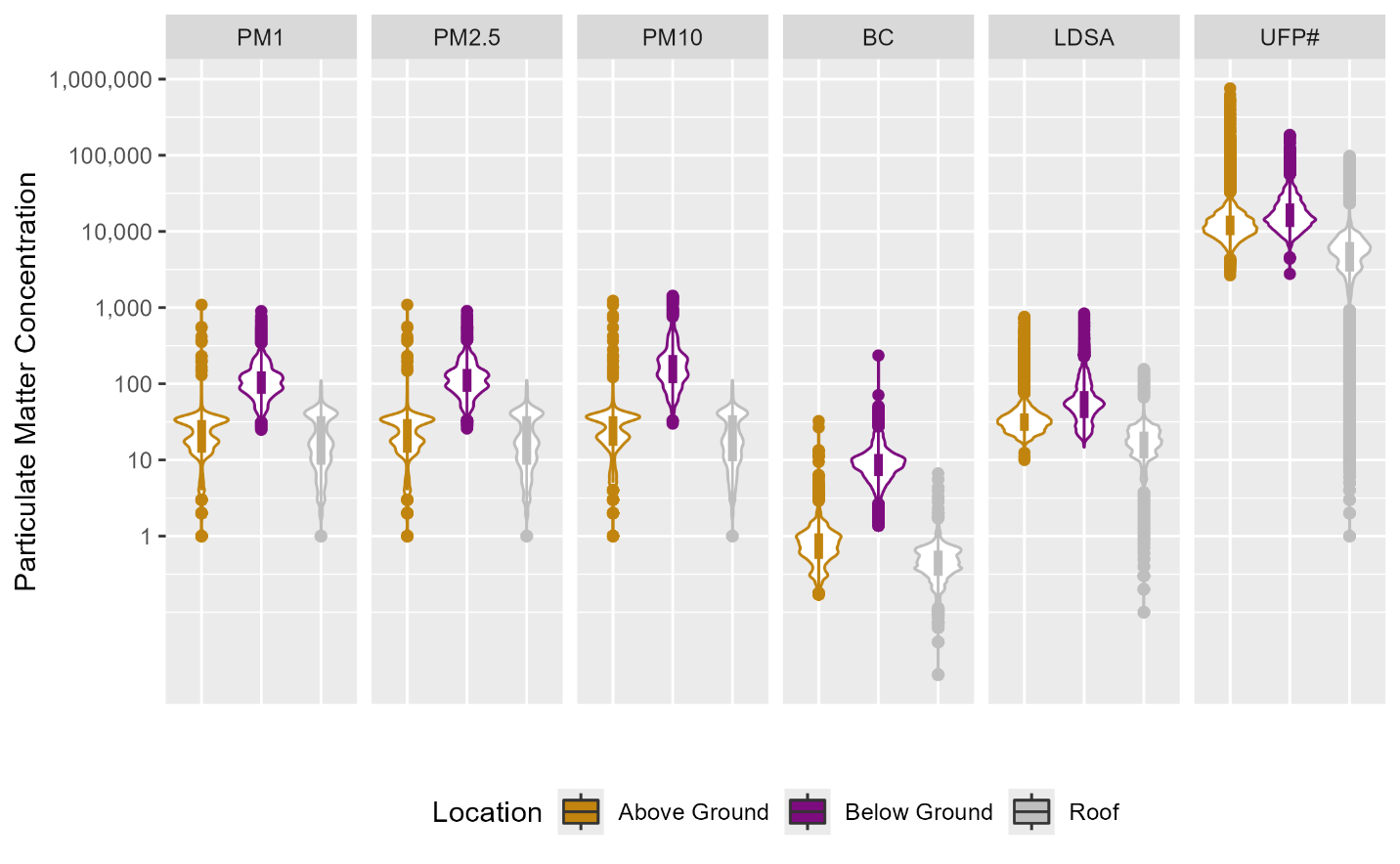


Figure S4. PM, BC, and UFP concentrations at aboveground (AG), belowground (BG), and suburban location (ROOF). The box shows the interquartile range with median value for each location, whereas the violin pattern outside shows the entire pollutant distribution. The figure is presented in a log scale with units µg/m^3^ for PM_1_, PM_2.5_, PM_10_ and BC, µm^2^/cm^3^ for UFP LDSA, and particle/cm^3^ for UFP number concentration (UFP#). The dots show the outliers near both above and below the whiskers.

Table S1. Pollutant concentration at belowground (BG) and aboveground (AG) locations for both separate sampling days and all sampling days together during July 2022

| Date | Location | Concentration  Mean ± Standard Deviation | | | | | |
| --- | --- | --- | --- | --- | --- | --- | --- |
|  |  | PM_1_  (µg/m^3^) | PM_2.5_  (µg/m^3^) | PM_10_  (µg/m^3^) | BC  (µg/m^3^) | UFP (LDSA)  (µm^2^/cm^3^) | UFP (Number)  (particle/cm^3^) |
| 7/13  Wed | BG | 170.8±63.1 | 182.2±67.8 | 304.8±150.5 | 14.5±5.9 | 105.6±45.3 | 27761±11518 |
|  | AG | 19.4±7.9 | 19.8±8.1 | 23.8±21.2 | 0.6±0.4 | 27.8±19.9 | 11095±13256 |
| 7/15  Fri | BG | 64.3±39.7 | 67.5±39.9 | 80±40.4 | 5.1±2 | 29±17.5 | 12746±7560 |
|  | AG | 15.2±9.3 | 15.4±9.4 | 19.8±10.9 | 0.8±0.5 | 44.8±20.4 | 16239±5847 |
| 7/18  Mon | BG | 128.8±63.9 | 138.6±68.9 | 231.9±173.1 | 8.8±2.3 | 53.9±14.7 | 16966±5189 |
|  | AG | 29.6±3.8 | 30.3±3.9 | 34.3±4.1 | 0.9±1.1 | 35.9±21.3 | 15885±17773 |
| 7/20  Wed | BG | 111.8±59.5 | 121.2±63.4 | 187.9±105.4 | 8.5±2.6 | 58.6±26.1 | 16884±6523 |
|  | AG | 36.4±7.7 | 36.8±7.7 | 39.9±8.4 | 1.2±1 | 38.4±13.6 | 14465±7773 |
| 7/22  Fri | BG | 113.6±38 | 120.8±39.4 | 163.4±53.7 | 13.5±7.4 | 74.2±40.6 | 18566±8934 |
|  | AG | 4.6±6.8 | 4.9±7.2 | 8.5±9.6 | 0.8±0.6 | 27.4±26.8 | 27761.2±11518.3 |
| Mean | BG | 112.2±61.3 | 120.0 ±65.5 | 182.1±132 | 9.5±5.4 | 59.4±36.2 | 17505±8685 |
|  | AG | 21.8±13.8 | 22.2±13.9 | 26.0 ± 15.7 | 0.9±0.8 | 35.8±21.7 | 14216±14592 |

Table S2. Real-time and gravimetric mean for PM_2.5_ with temperature and humidity for five different sampling days for aboveground and belowground

| Date | Real-time PM_2.5_  (DustTrak)  Mean ± Standard Deviation  (µg/m^3^) | | Gravimetric mean PM_2.5_  (Filter) (µg/m^3^)  Mean ± Standard Deviation  (µg/m^3^) | | DustTrak to Gravimetric Ratio | | Temperature (mean) °C | | Relative Humidity (mean)  % | |
| --- | --- | --- | --- | --- | --- | --- | --- | --- | --- | --- |
|  | BG | AG | BG | AG | BG | AG | BG | AG | BG | AG |
| 7/13/2022 | 182.2 ± 67.8 | 19.8 ± 8.1 | 102.3 ± 9.1 | 11.7 ± 3.5 | 1.78 | 1.71 | 25.1 | 24.3 | 49.4 | 50.3 |
| 7/15/2022 | 67.5 ± 39.1 | 15.4 ± 9.4 | 54 ± 2 | 15.7 ± 3.1 | 1.25 | 1.02 | 28.9 | 26.6 | 42.6 | 36.6 |
| 7/18/2022 | 138.6 ± 68.9 | 30.3 ± 3.9 | 91 ± 0.2 | 16.1 ± 1.5 | 1.53 | 1.88 | 26.9 | 31.3 | 39.5 | 55.1 |
| 7/20/2022 | 121.2 ± 63.4 | 36.8 ± 7.7 | 98 ± 4.5 | 28 ± 2.7 | 1.24 | 1.31 | 27.2 | 25.6 | 49.9 | 42.9 |
| 7/22/2022 | 120.8 ± 39.4 | 4.9 ± 7.2 | 99.2 ± 4 | 16.9 ± 1.5 | 1.22 | 0.29 | 22.1 | 26.5 | 53.3 | 38 |

# Comparison between gravimetric and DustTrak aerosol monitor

The DustTrak monitor is a real-time optical aerosol monitor based on the detection of light scattered by the particles to its optic chamber ^1–3^. Therefore, the measurement may be influenced by the type of particles. To evaluate the DustTrak aerosol monitor measurement method, the measurements were compared with the gravimetric method of total PM_2.5_ mass collected on the filter. PM_2.5_ measured by DustTrak aerosol monitor was highly correlated with the gravimetric method at the belowground location (r = 0.83, p<0.05) and moderately correlated at the aboveground location (r = 0.58, p<0.05) (Figure S3). The PM_2.5_ masses collected by DustTrak mostly fall above the line of unity, meaning the DustTrak measurements of PM_2.5_ mass were greater than the gravimetric method for both locations (except July 22^nd^, 2022, aboveground) (Figure S3). There was one anomaly for 7/22 when dusttrak measurements were showing very low measurements compared to all other days.

The mass concentration of PM measurement based on a light scattering principle strongly depends on the particle size distribution, shape and composition of the particles ^4–6^, which implies the relationship between the two methods may vary. PM_2.5_ measured by the DustTrak monitor compared to the gravimetric method was 1.4 times higher belowground and 1.2 times higher aboveground (Table S2). The greater difference belowground than aboveground suggests a correction for optical measurement to be more important for belowground measurements than the aboveground street-level environment. Using different real-time instruments (pDR 1500), which also uses similar light-scattering technology as DustTrak, previous studies in subway systems reported, gravimetric mean PM_2.5_ concentrations were 2 to 3-fold greater ^7^ and 1.5 to 4-fold greater ^8^ than the real-time PM_2.5_ concentration. In contrast, while comparing PM_2.5_ exposures of a group of boilermakers to wielding fumes using DustTrak and gravimetric methods, Kim et al. ^9^ found the mean concentrations of the methods were similar (DustTrak= 0.58 mg/m^3^, gravimetric= 0.54 mg/m^3^) and moderately to highly correlated (r= 0.68). The authors attributed the similar concentrations of both real-time and gravimetric method to the fact that the sampling location had a similar source for PM_2.5_ (wielding fume and fly ash) ^9^. Since the source of belowground PM_2.5_ can be from both inside the subway station and outside road traffic, the mean PM_2.5_ concentration belowground measured by the DustTrak monitor compared to the gravimetric method was 1.4 times higher in belowground whereas in the aboveground was 1.2 times higher (Table S2).

Previous studies reported that relative humidity greater than 80% can also affect the light scattering ability of the particles ^3,10^. However, for our sampling days, the relative humidity ranged from 36.6% to 53.3% with varying temperatures (Table S2).

Since there was variability in the measurements of different days (Figure 3) and also in both the DustTrak and gravimetric PM_2.5_ concentrations (Figure S3), to avoid additional bias or uncertainty, we did not correct the data. We presented the comparison between the concentrations from two different methods without adjustments to provide an unbiased and clearer comparison between the two measurement methods.

**References**

1 TSI Inc. DustTrak DRX Aerosol Monitor Model 8533/8534/8533EP. 2021.https://tsi.com/getmedia/3699890e-4adf-452f-9029-f3725612d5d1/8533-8534-DustTrak_DRX-6001898-Manual-US?ext=.pdf.

2 Javed W, Guo B. Performance Evaluation of Real-time DustTrak Monitors for Outdoor Particulate Mass Measurements in a Desert Environment. *Aerosol Air Qual Res* 2021; **21**: 200631.

3 Wang Z, Calderón L, Patton AP, Sorensen Allacci M, Senick J, Wener R *et al.* Comparison of real-time instruments and gravimetric method when measuring particulate matter in a residential building. *Journal of the Air & Waste Management Association* 2016; **66**: 1109–1120.

4 Hinds WC, Zhu Y. *Aerosol Technology: Properties, Behavior, and Measurement of Airborne Particles*. John Wiley & Sons, 2022.

5 Rivas I, Mazaheri M, Viana M, Moreno T, Clifford S, He C *et al.* Identification of technical problems affecting performance of DustTrak DRX aerosol monitors. *Science of The Total Environment* 2017; **584–585**: 849–855.

6 Zhang J, Marto JP, Schwab JJ. Exploring the applicability and limitations of selected optical scattering instruments for PM mass measurement. *Atmospheric Measurement Techniques* 2018; **11**: 2995–3005.

7 Vilcassim MJR, Thurston GD, Peltier RE, Gordon T. Black Carbon and Particulate Matter (PM2.5) Concentrations in New York City’s Subway Stations. *Environ Sci Technol* 2014; **48**: 14738–14745.

8 Luglio DG, Katsigeorgis M, Hess J, Kim R, Adragna J, Raja A *et al.* PM2.5 Concentration and Composition in Subway Systems in the Northeastern United States. *Environmental Health Perspectives* 2021; **129**: 027001.

9 Kim JY, Magari SR, Herrick RF, Smith TJ, Christiani DC, Christiani DC. Comparison of Fine Particle Measurements from a Direct-Reading Instrument and a Gravimetric Sampling Method. *Journal of Occupational and Environmental Hygiene* 2004; **1**: 707–715.

10 Wallace LA, Wheeler AJ, Kearney J, Van Ryswyk K, You H, Kulka RH *et al.* Validation of continuous particle monitors for personal, indoor, and outdoor exposures. *J Expo Sci Environ Epidemiol* 2011; **21**: 49–64.
